# Zika: what we do and do not know based on the experiences of Brazil

**DOI:** 10.4178/epih.e2016023

**Published:** 2016-05-31

**Authors:** Cristina Possas

**Affiliations:** Bio-Manguinhos, Oswaldo Cruz Foundation, Rio de Janeiro, Brazil

**Keywords:** Zika virus, Microcephaly, Guillain-Barré syndrome, Epidemiology, Immunity, Pathogenesis

## Abstract

**OBJECTIVES:**

Zika virus, which was first discovered in 1947, has become a global threat to human health as it is rapidly spreading through Latin America, the Caribbean, the US and Asia, after causing a large outbreak in the Northeast region of Brazil in 2015. There is ample evidence to support that Zika virus is associated with neurological complications such as microcephaly. The review aims to provide an overview on the complex issues involved in the emergence of Zika virus’s neurological disorders and to discuss possible explanations of Zika virus introduction and dissemination in Brazil. We also suggest national and global strategies to adequately respond to the Zika virus emergence.

**METHODS:**

We provide an analytical evaluation of the main issues related to the Zika outbreak in Brazil, based on available scientific literature, including government documents, and on epidemiological information from national surveillance databases.

**RESULTS:**

The studies on the clinical manifestations of the Zika virus infection coupled with the epidemiological surveillance information in Brazil have provided significant evidence that the Zika virus is associated with neurological disorders such as microcephaly and Guillain-Barré syndrome. Based on phylogenetic and molecular analysis, the hypothesis regarding the introduction of Zika virus in the country is that it took place following international events in 2013 and 2014, when many foreign visitors could have brought Zika virus into Brazil. The immunologically naïve status of populations in the Americas, previous infection with dengue virus, and the increased activity of *Aedes aegypti* might be the contributing factors for such an outbreak in Brazil. The Zika virus emergence emphasized the importance of cross-disciplinary perspective. Besides the scientific-based vector control strategies, it is important to understand the nature of the evolutionary processes involved in the viral evolution in complex ecosystems and to have social and anthropological knowledge on the conditions related to the spread of the disease in order to properly respond to the spread of the Zika virus.

**CONCLUSIONS:**

The experiences of Brazil have demonstrated the significance of multi-disciplinary approach in response to new and resurgent arboviral diseases and provided important lessons that could be applied to other developing countries.

## INTRODUCTION

Zika virus was first discovered in Uganda in 1947, and the first human case was documented in 1952 [1]. Before 2015, the virus was limited to Africa, Southeast Asia, and the Pacific Islands. However, since the report of the first autochthonous case of Zika virus infection in Brazil in May 2015, Zika has begun to spread to other countries in Latin America and the Caribbean, as well as to the US. Following the establishment of the a surveillance protocol for Zika virus infection-related microcephaly by the Brazilian National Response Plan, the Pan American Health Organization (PAHO) issued an epidemiological alert on January 17, 2016 regarding an association of Zika virus infection with neurological syndrome and congenital malformations [2]. Subsequently the World Health Organization declared Zika to be a public health emergency of international concern on February 1, 2016 [3]. The Emergency Operations Center of the US Centers for Disease Control and Prevention (CDC) was also activated, with a level 1 activation announced on February 8, 2016. A recent review study leaded by CDC investigators concluded that a causal relation can be inferred and the Zika virus can cause microcephaly if a woman is infected during pregnancy [4].

This paper will examine the complex issues involved in the emergence of Zika virus and discuss hypotheses regarding its introduction and dissemination in Brazil. Additionally, the paper also aims to provide insights into the eco-social determinants of Zika virus emergence and ways of integrating diverse conceptual frameworks into a transdisciplinary perspective. We hope that the lessons learned from the Brazilian experience can provide valuable insights for dealing with the emergence of new infectious diseases in other developing countries.

## WHAT WE KNOW

### Epidemiology

Zika virus is an arbovirus in the *Flavivirus* genus and is mainly transmitted through the bite of infected *Aedes* mosquitoes (https://commons.wikimedia.org/wiki/File:Aedes_aegypti_feeding.jpg), which are also vectors for chikungunya, dengue, and yellow fever [5].

The symptoms of Zika virus infection are mild, similar to those of dengue, including fever, rash, joint pain, muscle pain and/or conjuntivitis and in most cases hospitalization is not required [6]. However, the virus, which had been confined for six decades to ecological niches in tropical and subtropical areas in Africa and Asia, has changed its behavior from causing mild illness in a limited number of human cases to causing severe neurological disorders in newborns (microcephaly) and adults (Guillain-Barré syndrome) in Brazil.

### Virus

Two lineages of the Zika virus exist: the African lineage and the Asian lineage [7]. The virus involved in the outbreak in Brazil and in the Americas has been found to be the Asian-lineage virus, which was isolated in French Polynesia in 2013 to 2014 [8]. The full genome of the Zika virus has been published in Brazil and is being further analyzed. No evidence has currently been found that the Zika virus has undergone significant mutations that could be related to changing patterns of virus spread and pathogenesis [9-11].

### Neurological complications

**Figure 1.**
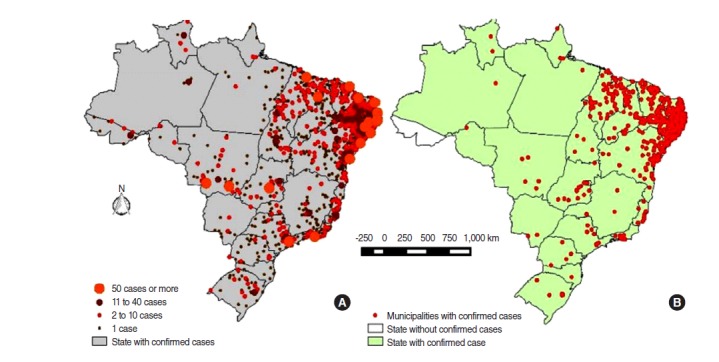
Distribution of notified and confirmed cases of microcephaly and/or central nervous system alterations. Cumulative cases by epidemiological week 19, 2016. Zika virus cases in Brazil: 7,534 notifications in 1,407 municipalities (A), and 1,384 confirmed cases in 499 municipalities (B), 427 of 499 (85.6%) of municipalities located in Northeast region. From: Ministry of Health, Brazil; Center for Emergency Operations in Public Health on Microcephaly (COES-Microcephalies). Epidemiological informs 1/2015 to 19/2016: monitoring microcephaly cases in Brazil, 2016 [13].

Severe fetal birth defects such as microcephaly have been found in infants born to women infected with Zika virus, according to PAHO guidelines and National Surveillance Protocol in Brazil [12,13]. The average number of reported cases of microcephaly in newborns in the years before the emergence of Zika virus in Brazil in May 2015 was 200. Recently, the number of cases has increased exponentially, and the incidence of microcephaly cases in 2015 has been reported to be 20 times higher than in previous years [9]. In February 2016, the Brazilian Ministry of Health reported 5,640 cases of microcephaly, most of them Zika virus infection-related, with 4,107 cases under investigation. Of these cases, 538 were confirmed, with 120 deaths, and as Figure 1 shows, most were concentrated in the economically disadvantaged northeast area of the country (93.6%) [13].

Subsequently, several scientific studies have been conducted regarding Zika virus infection-related neurological disorders in newborns [14]. In one study, the authors were able to determine the complete sequence of Zika virus from a microcephalic fetus that was aborted by a Slovenian mother who visited the northeast region of Brazil and showed symptoms of Zika virus infection, including fever and rash [15]. At approximately the same time, researchers in Brazil and at the US CDC found Zika virus in the brains of two newborn babies who died shortly after birth [16].

Additionally, an increasing number of Zika virus infection-related cases of Guillain-Barré syndrome have been observed in Brazil. Although significant under-reporting may be expected to take place because Guillain-Barré syndrome is not a notifiable disease, information from Brazilian hospitals indicates an increase of 29.8% in hospitalizations from 2014 to 2015, from 1,439 to 1,868 cases; of note, some hospitals, such as Antonio Pedro Hospital in Rio de Janeiro, have reported an unusual increase in the number of cases (16 cases in January 2016, reflecting a more severe caseload than usual).

## WHAT WE DO NOT KNOW

### Pathogenesis and transmission

The pathogenesis and the mechanisms by which the Zika virus exerts its potentially severe effects on the brain of newborns and adults are still unknown. In Brazil, complete information regarding the number of pregnant women infected by Zika virus who gave birth to babies with microcephaly is lacking due to significant under-reporting and limited access to health care in the poorest regions. Although studies have suggested that Zika virus infection in the first trimester of pregnancy is related to more severe cases of microcephaly in newborns, a recent groundbreaking study in Brazil has found cases of Zika virus-associated severe microcephaly in all stages of gestation, including cases in the second and third trimester of pregnancy [14]. We do not know yet how the effect of Zika virus differs depending on the time of the infection during pregnancy. Some evidence suggests that Zika virus has the ability to access the central nervous system through enhanced blood-brain barrier permeability and to cause neurological disorders. However, the definitive mechanisms involved in the neuroinvasiveness of Zika virus are still unclear and further studies are needed. Additionally, it is necessary to further clarify the patterns of severe brain damage and other developmental problems that may be caused by Zika virus infection during pregnancy, such as unformed parts of the newborn brain, calcifications, and other neurological and ocular pathologies [17,18]. Many possibilities regarding transmission likewise remain uncertain. Concerns have been raised that Zika virus could adapt to the *Aedes albopictus* mosquitoes that are widespread in Latin and Central America, the US, and other regions not yet affected by Zika virus [19]. Furthermore, entomological studies have recently suggested that *Culex*, a tropical domestic mosquito widespread in Brazil, might be able to transmit Zika virus since some arboviral infections are transmitted by several species of *Culex* [20]. This possibility is now under investigation.

Additionally, other routes of transmission in addition to mosquito bites should be further studied. It is very likely that Zika virus can be spread through blood transfusions, and suspected cases in Brazil are under investigation. Sexual transmission has been confirmed by the US CDC as a route of transmission, although we do not know the transmission rate [21,22]. Although no reports have yet been published of infants becoming infected with Zika virus through breastfeeding, the follow-up of exposed children is recommended. Researchers at Oswaldo Cruz Foundation (Fiocruz) have identified Zika virus in saliva, but no evidence indicates that the concentration found is sufficient for oral transmission [23].

### Hypothesis on Zika virus introduction

A recent phylogenetic and molecular analysis by Brazilian researchers with international collaboration showed a single introduction of Zika virus into the Americas, estimated between May and December 2013 which indicates that the virus had been circulating in Brazil earlier than the first Zika virus infection case reported in 2015 [24,25]. This provides a possible link between international events which happened in 2013 and the introduction of Zika virus into Brazil, causing the outbreak. Three hypotheses, all related to international travel, have been proposed regarding the introduction of Zika virus to Brazil. These hypotheses include the visit of the Pope, World Youth Day in July 2013, the World Cup in 2014, and the canoeing championship in Rio de Janeiro in 2014. In 2013, Pope Francis visited Rio de Janeiro after his nomination, and many young Catholics from Africa and Asia visited Brazil, staying in poor local slums where *Aedes* mosquitoes tend to concentrate. In 2014, the World Cup could have introduced Zika virus to Brazil, since many foreigners gathered in stadiums located in multiple states in Brazil. Finally, teams of rowers from French Polynesia, where a Zika virus outbreak occurred in 2013 and 2014, attended a canoeing championship in Rio de Janeiro in 2014. Concerns have been raised that the Olympic Games may lead to the introduction of new viruses into the country and to the possible global dissemination of Zika virus and other infectious diseases, despite significant governmental efforts to minimize these risks.

Several explanations have been proposed regarding why the Zika virus emerged and caused an outbreak in Brazil. One possible explanation is the immunologically naive status of populations in the Americas compared to the populations of African and Asian countries where Zika virus has been circulating for decades and widespread immunity most likely exists against the virus [24,25]. A second reason may be that aggravated Zika virus symptoms and severe sequelae are specifically observed in patients who have been previously infected with one or more of the four dengue viruses present in Brazil, as it has been suggested that Zika virus could build on previous exposure to dengue [26]. The latter hypothesis is supported by the fact that a significant increase in dengue cases has been observed, from 48,875 in January 2015 to 73,872 cases in the same period in 2016. This increase in the number of dengue cases may reflect not only a 48% increase in dengue infections specifically, but also an increase in *Aedes aegypti* mosquito activity. Additionally, it is important to note that during the large outbreak in Brazil, as in French Polynesia, the symptoms of Zika virus were reported in the context of a concurrent dengue epidemic [4]. It is also possible that the Zika outbreak in Brazil went unnoticed for some time because dengue and chikungunya, which are spread by the same species of *Aedes* mosquitoes, produce more serious symptoms, such as higher fever and worse pain.

### Vector control strategies

For several political and institutional reasons, related to the decentralization of responsibilities in the National Health System and the elimination of federal structures in charge of vector control, such as the Superintendency of the Public Campaigns of Brazil - SUCAM in 1990, routine vector control has not been treated as a high priority in Brazil, although successful efforts have been made in some states and municipalities. In this context, emergency strategies are now under evaluation. In order to deal with Zika virus infection, several novel vector control strategies have been recently proposed and are now under the scrutiny of the scientific community and policy makers. One strategy is to modify the genes of mosquitoes to produce faulty offspring and reduce the quantity of larvae. This strategy has already been approved by the National Biosafety Commission in Brazil and applied by a British company, Oxitec, in some Brazilian cities. Another strategy is the sterile insect technology developed by the United Nations International Atomic Energy Agency, in which laboratory-bred male mosquitoes are exposed to nuclear radiation in order to make their sperm sterile before being released into the wild to mate with female mosquitoes. In this way, the mosquito population could be reduced in a few months. Another promising strategy to control Zika virus transmission by mosquitoes is using *Wolbachia* bacteria as a method of biologically controlling *Aedes* mosquitoes [27]. Nevertheless, these vector control strategies are still experimental and their long-term consequences to the environment are still not clear. In addition, another recent strategy, adopted in the US and now under evaluation in some cities in Brazil, is to use drones as a technology for locating the breeding sites of mosquitoes, allowing pesticides to then be spread in a more specific and environmentally friendly way.

### Knowledge, policies, and interventions: the need for a cross-disciplinary perspective

The Zika virus outbreak in Brazil revealed the need to further develop two important areas of knowledge in controlling and managing this arbovirus. The first crucial area of knowledge is the ecosystem. Having a comprehensive ecosystem-focused perspective is crucial in addressing vector-borne diseases with complex dynamic cycles involving vertebrates and blood-feeding vectors, such as mosquitoes and ticks. The importance of an ecological perspective is often missed in public health and epidemiological surveillance, and the absence of this perspective might explain previous failures in dealing with unexpected pandemics. The complex conditions affecting the evolution, introduction, mutations, variations, and adaptations of arboviruses in new ecological niches should be understood from this perspective [28-30]. In particular, a variational approach [29] to evolution is a key for understanding disease emergence because it provides insight into the evolutionary changes in vectors, pathogens, and diseases [28]. Additionally, it is important to understand that evolution is not progressive; rather, it occurs in opportunistic and unpredictable ways. The notions that usually prevail in global public health and epidemiological surveillance approaches include equilibrium-oriented points of view, assumptions of linearity, and teleological perspectives. However, these notions must be overcome to deal with disease evolution in complex ecosystems and with unpredictable evolutionary processes in general [28-30].

Social and anthropological issues are another important area. Although genetic, immunological, and environmental conditions are important, understanding the social, economic, and anthropological aspects of the vulnerable population is also necessary because a pathogen requires a receptive population in order to cause disease. The specific social issues contributing to the Zika virus outbreak include rapid urbanization in conditions of extreme poverty, leading to intense deforestation that favors the contact of populations with unknown vectors and pathogens, as well as the intensification of international travel and population mobility. In addition, social vulnerabilities to new disease are not only a consequence of socioeconomic conditions, but also of the social behaviors related to risk and disease perceptions. It is therefore important to understand cultural and behavioral barriers when dealing with the emergence of new diseases. The importance of such barriers is demonstrated, for example, by the vulnerability of web users in Brazil to an erroneous rumor disseminated online that attributed Zika virus infection-related disease in newborns to vaccinations in pregnant women. Such barriers are also reflected by the social leniency of policy makers and the population overall regarding vector control measures, despite the availability of widespread information about this issue. Some studies have examined why access to information is not sufficient to change perceptions and behaviors. These studies have indicated that understanding the emotional component of attitudes is a crucial factor that could bring about effective access to behavior-changing information, and that an adequate understanding of emotions is required in the dissemination of new information [31,32]. These results support the conclusion that adopting an “emotional design of public health information” is likely to be crucial for changing behaviors and managing new emerging diseases such as Zika.

Other social and organizational issues in Brazil should be addressed in response to the Zika virus. Concerning health policy, the overall quality of National Health System services should be improved to deal with outbreaks of Zika virus. Although Brazil has a well-established universal health care system, it has been severely underfunded and undermined by the rapid increase of low-quality private health plans. Additionally, despite the existence of a well-established epidemiological surveillance system in Brazil, surveillance programs should be strengthened and refined in order to improve the quality of indicators and the ability to respond to unexpected occurrences of new and resurgent diseases.

Another issue is social policy concerning poverty and inequality. Although nearly everyone in Brazil is now exposed to *Aedes* mosquitoes and to Zika as well as dengue and chikungunya, the poorest tend to be more vulnerable, and nearly 94% of Zika virus-related microcephaly cases have been reported in the economically deprived northeast region of Brazil. The majority of the Brazilian population lives in poor areas without adequate housing and sanitation, meaning that policies for better housing, sanitation, and garbage collection should be urgently implemented.

The specific nature of the disease caused by Zika virus also highlights certain ethical issues. Abortion in Brazil is illegal; therefore, when effective diagnostic kits for Zika virus infection are developed, Brazilian society will have to come to a consensus regarding what to do when a pregnant woman tests positive for Zika infection. Women’s movements and organizations advocating for reproductive rights may be expected to play a crucial role in this process.

Moreover, environmental policies are important in light of the dramatic global environmental and climate changes related to accelerated deforestation, urbanization, and global warming; these changes affect Brazilian ecosystems and have favored the proliferation of vectors and the emergence of new infectious diseases. Although the Brazilian government has put in place an extensive national campaign to mobilize the population to avoid the proliferation of *Aedes* in their houses as a response to the Zika virus outbreak, this campaign has not been effective because it has not been able to address transmission outside the home, which is conditioned by the urban and environmental determinants of transmission. Novel Zika virus control methods that incorporate environmental, urbanization, social, sanitation, and vector control policies with broader perspectives are urgently needed.

Innovations and technological development policies focused on the development of new products are a major aspect of dealing with the Zika virus outbreak in Brazil. Currently, Brazilian institutions, despite very limited resources for research and development, are working on developing new and innovative diagnostic tools for the Zika virus, and it is expected that a new diagnostic tool produced by Bio-Manguinhos/Fiocruz will be available soon, after registration with the Federal Government Agency. This Bio-Manguinhos product is an ‘on-the-spot’ rapid test kit for measuring immunoglobulin M and immunoglobulin G.

## CONCLUSION

The experiences of Brazil with Zika virus underscore the conclusion that the conceptual approach to this new disease should shift from a traditional tropical neglected disease framework to an emerging and resurgent neglected disease perspective. Emerging and resurgent neglected diseases such as Zika virus are no longer confined to tropical regions and the most economically disadvantaged areas of the globe, but threaten other countries, including developed nations such as the US and European countries. Vector-borne viral diseases therefore deserve more public health attention, and an effective eco-social approach is needed in order to anticipate risks and provide interventions before the emergence and global spread of new and resurgent diseases. The experiences of Brazil have demonstrated that a cross-disciplinary approach supporting a social ecosystem health perspective [30] is crucial for anticipating future global scenarios and for developing effective interventions to deal with new and resurgent arboviral diseases.
